# Supplementary material for: Work for self or others? Two different kinds of burnout in China
Source: PLoS One. 2025 Nov 11;20(11):e0334394. doi: 10.1371/journal.pone.0334394 (PMC12604786; doi:10.1371/journal.pone.0334394)
Supplement: S1 Appendix — (DOCX) [file pone.0334394.s001.docx]

**Appendix**

Original Survey Questions and Options

| Variable Group | Occupational Group | Variable  Name | Original  Questionnaire  Number | Question | Unique Values  / Numeric |
| --- | --- | --- | --- | --- | --- |
| JR | EM | union_help | I3a1_17_3 | To what extent does the labor union in your unit/enterprise provide assistance? | Yes; No |
|  |  | payment_level | I3a1_4 | What is the current method of salary calculation for your job? | Monthly Salary; Daily Calculation; Performance-based or Base Salary plus Performance; Hourly; Annual Salary; Commission or Base Salary plus Commission; Piece Rate; Sometimes Piece Rate, Sometimes Hourly; Other |
|  |  | position_security | I3a1_8 | What type of labor contract have you signed? | Permanent Contract; Fixed-term or Short-term Contract |
|  |  | content_control | I3a_20_1_w16 | To what extent are the contents of your work tasks determined by yourself? | Completely Determined by Yourself; Completely Determined by Others; Partially Determined by Yourself |
|  |  | schedule_control | I3a_20_2_w16 | To what extent are the scheduling arrangements of your work determined by yourself? | Completely Determined by Yourself; Completely Determined by Others; Partially Determined by Yourself |
|  |  | intensity_control | I3a_20_3_w16 | To what extent are the workload/intensity of your work determined by yourself? | Completely Determined by Yourself; Completely Determined by Others; Partially Determined by Yourself |
|  |  | position_level | I4a_2_8 | What is your main occupation in your current/latest employment? | General Staff; Middle-level Business Manager; General Business Manager; Business Leader; Middle-level Party Affairs Manager; Middle-level Administrative Manager; Administrative Leader; General Administrative Staff; Party Affairs Leader; General Party Affairs Staff |
|  | SE | entre_reason | I3a3_1 | Was this entrepreneurial venture started because of finding a good business opportunity or due to lack of better job options? | Both Reasons; Lack Better Job Options; Seized Good Opportunity; Had Good Jobs but Chose Entrepreneurial Opportunity |
|  |  | entre_channel | I3a3_2_1_w16 | Which channel did your business opportunity come from? | Self-discovery; Introduced by Friends; Previous Work Experience; Imitation of Other Enterprises Nearby; Others |
|  |  | gov_customers | I3a3_8_1 | Is your business relationship with government agencies/staff stable? | Very Stable; Quite Stable; Not Very Stable; Unstable |
|  |  | institution_clients | I3a3_8_2 | Is your business relationship with institutional staff/staff stable? |  |
|  |  | corporate_clients | I3a3_8_3 | Is your business relationship with corporate entities/staff stable? |  |
|  |  | social_organization | I3a3_8_4 | Is your business relationship with social organizations/entities stable? |  |
|  |  | individual_customers | I3a3_8_5 | Is your business relationship with individual customers stable? |  |
|  |  | busi_channel | I3a3_16s | [Multiple Choice Count] Did your first year's business involve any of the following channels: Government and Party Organs, State-owned Enterprises, Public Institutions, Collective Enterprises, Individual Businesses, Private Enterprises, Foreign-invested/Joint Ventures, Shareholding Enterprises, Individuals/Customers, or Overseas Enterprises? | 1; 0; 2; 3; 9; 8; 4; 5; 6 |
|  |  | provided_business | I3a3_17 | How many people proactively offered business opportunities when you started your business? | numeric |
|  |  | provided_business_known | I3a3_18 | Did you know the person who provided the most important business opportunity before? | Yes; No |
|  |  | provided_business_num | I3a3_19 | Approximately how many people have introduced business to you so far? | numeric |
|  |  | provider_department | I3a3_21s | [Multiple Choice Count] Among those who introduced business to you, were there any working in the following departments: Government and Party Organs, State-owned Enterprises, Public Institutions, Collective Enterprises, Individual Businesses, Private Enterprises, Foreign-invested/Joint Ventures, Shareholding Enterprises, Federation of Industry and Commerce, Entrepreneurs Association, Industry Associations or Professional Associations? | 1; 0; 3; 2; 5; 6; 9; 4; 7; 12; 8; 11 |
|  |  | relation_nohelp | I3a3_22_1 | Found connections but they didn't help much? | True; False |
|  |  | relation_nodeci | I3a3_22_2 | Some connections helped, but not decisively? |  |
|  |  | relation_onlykey | I3a3_22_3 | Only one connection played a decisive role? |  |
| JD | EM | wage_delay | I3a1_18_1_1_ | Need to endure wage arrears | No; Not applicable; Yes |
|  |  | extra_work | I3a1_18_1_2_ | Need to endure mandatory overtime |  |
|  |  | injury | I3a1_18_1_3_ | Need to endure work-related injuries |  |
|  |  | unsafety | I3a1_18_1_4_ | Need to endure safety protections at work that do not meet national standards |  |
|  |  | pollution | I3a1_18_1_5_ | Endure workplace pollution levels exceeding standards |  |
|  |  | physical_labor | I3a1_19_1 | Need to endure heavy physical labor | Sometimes; Not applicable; Rarely; Never; Often |
|  |  | physical_move | I3a1_19_2 | Need to frequently change body positions while working |  |
|  |  | mental_labor | I3a1_19_3 | Requires quick response thinking or mental labor |  |
|  |  | internet_skill | I3a1_19_4 | Need to use the internet |  |
|  |  | need_training | I3a1_9 | Is specialized training or education necessary to perform this job well? | Not required; Not applicable; Required |
|  |  | skill_demand | I3a1_10 | How much time did you spend mastering the main skills required for this job? | More than one year; Not applicable; One to three months; More than three months but less than one year; One day; More than three years; Less than one month; Several days; About one week |
|  |  | min_education | I3a_23_w18 | What is the minimum educational level needed to be competent in your current job, in your opinion? | Junior high school; College; Bachelor's degree; High school/vocational high school; Doctorate; Not applicable; Master's degree; Elementary school |
|  |  | min_experience | I3a_24_w18 | In your opinion, how much work experience is needed to be competent in your job? | Less than one year; Three to five years; Six to ten years; One to two years; Not required; Not applicable |
|  | SE | skill_demand | I3a3_2_2_1_w16 | How important is having technical expertise (such as relevant research achievements or patents) to starting a business, in your view? | Average; Not applicable; Important; Very important; Unimportant; Extremely unimportant |
|  |  | exp_support | I3a3_2_2_2_w16 | How important is having business management experience to starting a business, in your view? |  |
|  |  | relation_support | I3a3_2_2_3_w16 | How important is having a network of contacts to starting a business, in your view? |  |
|  |  | eco_support | I3a3_2_2_4_w16 | How important is having an economic foundation to starting a business, in your view? |  |
|  |  | gov_support | I3a3_2_2_5_w16 | How important is government support to starting a business, in your view? |  |
|  |  | day_work_hours | I3a3_4_average | Average number of working hours per day | Numeric |
|  |  | month_rest_days | I3a3_6 | Average number of days off per month | Numeric |
|  |  | need_training | I3a3_9 | Is specialized training or education necessary to perform this job well? | Not required; Not applicable; Required |
|  |  | skill_demand | I3a3_10 | How much time did you spend mastering the main skills required for this job? | More than one year; Not applicable; One to three months; More than three months but less than one year; One day; More than three years; Less than one month; Several days; About one week |
|  |  | competition_start | I3a3_15 | Was the first year of your business particularly competitive? | Not very competitive; Not applicable; Extremely competitive; Not at all competitive; Quite competitive; No competition; Hard to say |
|  |  | competition_year | I3a3_24 | Has your business been particularly competitive since July 2017? |  |
| IC | FS | father_alive | I1_4_2 | Is your father still alive? | No; Yes; Not applicable |
|  |  | mother_alive | I1_5_2 | Is your mother still alive? |  |
|  |  | paternal_edu | I1_4_6 | Father's level of education | Junior high school; College; No schooling; Elementary/Private school; Regular high school; Not applicable; Others; Vocational high school; University bachelor's degree |
|  |  | maternal_edu | I1_5_6 | Mother's level of education |  |
|  |  | paternal_job | I1_4_9_w16 | Type of employer/father’s workplace | Self-employed (including registered self-employed or unregistered shop owners); State-owned enterprises; Not applicable; Government/collective public institutions; Farming: Agriculture, forestry, animal husbandry, sideline occupations and fishery production (such as farming, raising chickens, ducks, aquatic products, etc.); Village committees and other autonomous organizations; Collective enterprises; Private enterprises; Unfixed workers (scattered workers, vendors, nannies without dispatch units, self-employed drivers, manual craftsmen, etc.); Party and government organs, people's organizations, military; Non-governmental non-enterprise, social groups and other social organizations; Freelancers (internet writers, painters, media workers, freelance photographers, etc.) |
|  |  | maternal_job | I1_5_9_w16 | Type of employer/mother’s workplace |  |
|  |  | soeco_status | I7_10_4 | Where would you rank your family on a scale from 1-10 when you were 14 years old? | Numeric |
|  |  | spouse_cost | I1_2_3a_w18 | How much money did your spouse's family spend on your wedding? | Numeric |
|  |  | parent_cost | I1_2_4 | How much of your wedding expenses were covered by your elders? | Numeric |
|  | SS | job_hunt | I3d_4s | [Multiple Choice Count] What are the relationships of the people who have helped you in your job search process? | None/0; 1-3; 4-6; 7-9; More than 10 |
|  |  | tell_truth | I6_1_1 | Among close people in your area, how many can you confide your worries to? | Average; Very familiar; Not very familiar; Quite familiar; Completely unfamiliar |
|  |  | disc_imp | I6_1_2 | Among close people in your area, how many can you discuss important matters with? | Relatively trust; Average; Not very trusting; Very trusting; Extremely distrustful |
|  |  | borrow_money | I6_1_3 | Among close people in your area, how many could you borrow 5000 RMB from? | Relatively many; Few; A great deal; Average; Extremely few |
|  |  | familiarity | I6_3 | What is the extent of familiarity between you and other residents in your community/village? | Almost none/Less than 1 day; Seldom (1-2 days); Very much (often 3-4 days); Nearly constant (5-7 days) |
|  |  | mutual_trust | I6_4 | Do you trust your neighbors and other residents in your community/village? | No; Yes |
|  |  | mutual_help | I6_5 | Is there mutual assistance between you and your neighbors and other residents in your community/village? | No; Not applicable; Yes |
|  | MH | annoyed_trivial | I9_28_1_w16 | Being troubled by minor issues | Sometimes; Not applicable; Rarely; Never; Often |
|  |  | poor_appetite | I9_28_2_w16 | Loss of appetite |  |
|  |  | inner_distress | I9_28_3_w16 | Unable to shake off melancholy even with help from family and friends |  |
|  |  | low_self_esteem | I9_28_4_w16 | Feeling inferior to most others |  |
|  |  | concentration_issue | I9_28_5_w16 | Difficulty concentrating on tasks |  |
|  |  | low_mood | I9_28_6_w16 | Feeling emotionally down |  |
|  |  | task_effort | I9_28_7_w16 | Finding everything requires great effort |  |
|  |  | hopeless_future | I9_28_8_w16 | Feeling hopeless about the future |  |
|  |  | life_failure | I9_28_9_w16 | Believing one's life is a failure |  |
|  |  | fearful | I9_28_10_w16 | Feeling scared |  |
|  |  | poor_sleep | I9_28_11_w16 | Poor sleep quality |  |
|  |  | unhappy_feeling | I9_28_12_w16 | Feeling unhappy |  |
|  |  | speaks_little | I9_28_13_w16 | Talking less than usual |  |
|  |  | feels_lonely | I9_28_14_w16 | Feeling lonely |  |
|  |  | people_unfriendly | I9_28_15_w16 | Believing people are not very friendly towards oneself |  |
|  |  | life_meaningless | I9_28_16_w16 | Feeling life has no meaning |  |
|  |  | has_cried | I9_28_17_w16 | Having cried |  |
|  |  | sorrowful | I9_28_18_w16 | Feeling sorrowful |  |
|  |  | unliked_by_others | I9_28_19_w16 | Believing people dislike oneself |  |
|  |  | life_cant_continue | I9_28_20_w16 | Feeling unable to continue living |  |
|  | PH | smoke_self | I9_8 | Do you smoke? | Not required; Not applicable; Required |
|  |  | smoke_cowork | I9_9 | Are others smoking in your workplace? |  |
|  |  | drink | I9_10 | Do you drink alcohol? |  |
