# Supplementary material for: Work for self or others? Two different kinds of burnout in China
Source: PLoS One. 2025 Nov 11;20(11):e0334394. doi: 10.1371/journal.pone.0334394 (PMC12604786; doi:10.1371/journal.pone.0334394)
Supplement: Attachment — Submitted filename: response-2.docx [file pone.0334394.s004.docx]

To Dear Reviewer 1:

Thank you for your careful and rigorous review. We agree that your suggestions are very reasonable.

Following your advice, we have removed the statistical figures from the Discussion section:

- The p-values in 4.1 have been removed, as they are already indicated by asterisks (*) in the table in section 3.1, making further emphasis unnecessary.
- The data in 4.2 have been moved to section 3.2. At the beginning of the revised 4.2, we briefly restate the results from 3.2 and continue the discussion accordingly.
- The numbers in 4.3 have been entirely removed, as they were duplicated in the table in section 3.3 and thus redundant.
- Similarly, the numbers in 4.4 have been entirely removed, as they were duplicated in the table in section 3.4 and therefore unnecessary.
